# Near-IR-induced dissociation of thermally-sensitive star polymers†
†Electronic supplementary information (ESI) available. See DOI: 10.1039/c6sc04650a



**DOI:** 10.1039/c6sc04650a

**Published:** 2016-12-13

**Authors:** Yuqiong Dai, Hao Sun, Sunirmal Pal, Yunlu Zhang, Sangwoo Park, Christopher P. Kabb, Wei David Wei, Brent S. Sumerlin

**Affiliations:** a George & Josephine Butler Polymer Research Laboratory , Center for Macromolecular Science & Engineering , Department of Chemistry , University of Florida , PO Box 117200 , Gainesville , FL 32611-7200 , USA . Email: sumerlin@chem.ufl.edu ; Fax: +1 352 392 9741; b Department of Chemistry , Center for Nanostructured Electronic Materials , University of Florida , PO Box 117200 , Gainesville , FL 32611-7200 , USA

## Abstract

Responsive systems sensitive to near-infrared (NIR) light are promising for triggered release due to efficient deep tissue penetration of NIR irradiation relative to higher energy sources (*e.g.*, UV), allowing for spatiotemporal control over triggering events with minimal potential for tissue damage. Herein, we report star polymers containing thermally-labile azo linkages that dissociate during conventional heating or during localized heating *via* the photothermal effect upon NIR irradiation. Controlled release during conventional heating was investigated for the star polymers loaded with a model dye, with negligible release being observed at 25 °C and >80% release at 90 °C. Star polymers co-loaded with NIR-responsive indocyanine green showed rapid dye release upon NIR irradiation (*λ* ≥ 715 nm) due to the photothermally-induced degradation of azo linkages within the cores of the star polymers. This approach provides access to a new class of delivery and release systems that can be triggered by noninvasive external stimulation.

## Abstract 2

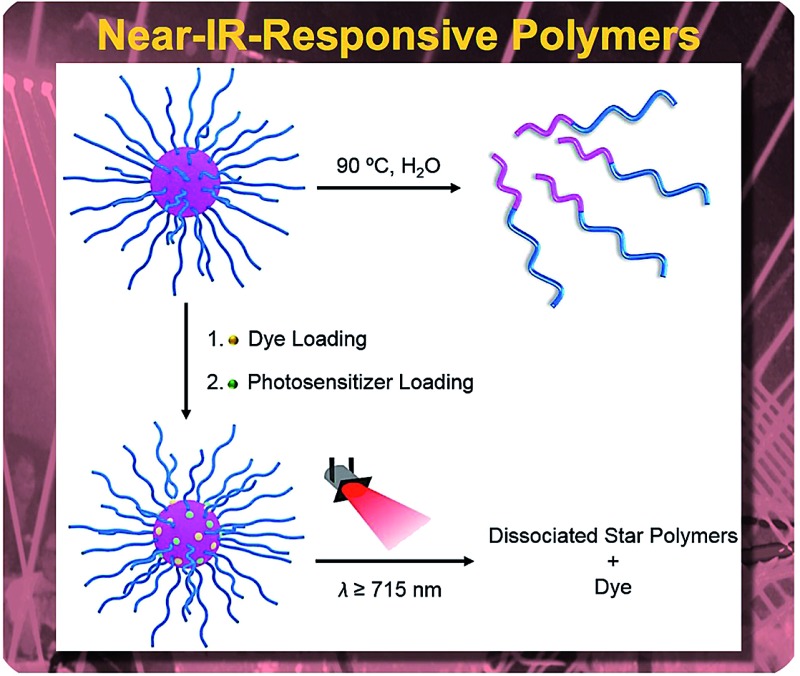
Responsive systems sensitive to near-infrared (NIR) light are promising for triggered release due to efficient deep tissue penetration of NIR irradiation relative to higher energy sources (*e.g.*, UV), allowing for spatiotemporal control over triggering events with minimal potential for tissue damage.

## Introduction

Developments in reversible–deactivation radical polymerization (RDRP) techniques, including atom transfer radical polymerization (ATRP),^1^ reversible addition–fragmentation chain transfer (RAFT) polymerization,^2^ and nitroxide-mediated polymerization (NMP),^3^ have opened access to remarkably diverse polymer architectures such as block, gradient, graft, hyperbranched, star, cyclic, and network polymers.^4^ Numerous examples of amphiphilic block copolymers have been reported over the last decade, with many of the copolymers possessing the ability to self-assemble into well-ordered nanostructures (*e.g.*, micelles and vesicles).^5^ Such block copolymer-based micelles can mimic the structure of plasma lipoproteins that form biological carriers, which has led to their extensive consideration as drug delivery systems.^6^ Generally, block copolymer micelles have a stabilizing hydrophilic shell and a hydrophobic core that entraps and protects drugs (*e.g.*, chemotherapeutics) from degradation. As micelle size increases, drug circulation times are extended and, in the case of cancer therapy, accumulation in tumor tissue can be achieved *via* the enhanced permeability and retention (EPR) effect.^7,8^ However, at low concentrations or in high ionic strength environments, the supramolecular nature of micelles often leads to thermodynamic instability and potential premature dissociation to unimers.^9^ Therefore, further stabilization of micelles is often needed to prevent premature disintegration and undesired drug release *in vivo*.^10,11^

As an alternative to micelles, stable and non-dissociable nanoparticles can be constructed through chemical crosslinking of supramolecularly associated block copolymers to yield core-crosslinked star polymers.^12–14^ In this approach, polymer assembly and stability is not only governed by hydrophobic interactions, but also by covalent bonding in the core. While studies have focused on star polymers that are permanently crosslinked,^15^ in many cases it is highly desirable to incorporate stimuli-responsive moieties within the nanocarriers to enable (external) triggerable dissociation. In this manner, the star polymers can serve as thermodynamically stable, covalently linked carriers until triggered by an appropriate stimulus. The degradation of crosslinked polymer assemblies also assists in the excretion of polymer residues after delivery, thereby preventing potential complications from prolonged circulation of non-degradable polymers.^16^ A variety of stimuli-responsive polymeric nanocarriers have been developed by incorporating (post-)cleavable bonding motifs such as disulfide,^17^ acetal,^18^ oxime,^19^ Diels–Alder,^20^ and light-labile linkages.^21^

Nanocarriers that are sensitive to electromagnetic irradiation are particularly advantageous as they could potentially provide remote (*i.e.*, external), on-demand, and spatiotemporal drug release by tuning the wavelength, power, and the irradiation site.^22^ Compared to UV and visible light, near-infrared (NIR) irradiation has unique advantages in drug delivery systems because it penetrates deeply through skin and underlying tissue without severly damaging healthy tissues.^23^ Combining NIR-responsive photosensitizers and thermally-degradable nanocarriers, targeted delivery can be achieved by employing local heat generated *via* the photothermal effect.

Generally, two approaches to photothermally-induced drug release are considered. The first involves controlling the phase transitions of thermally-sensitive polymeric carriers,^24^ such as those derived from poly(*N*-isopropylacrylamide). At temperatures above the cloud point, structural collapse of the carrier leads to the release of loaded drugs.^25^ The second method focuses on cleaving thermally-labile linkers between nanocarriers and active compounds, allowing the covalently encapsulated contents to be “unlocked”. Various photothermal nanoformulations have been investigated and utilized for on-demand drug release, including iron oxide nanoparticles,^26^ copper monosulfide nano-agents,^27^ carbon nanotubes,^28^ graphene,^29^ gold nanoparticles,^30,31^ and indocyanine green (ICG)-containing systems.^32–34^ Among these, ICG is a particularly promising component for photothermal therapy due to its low cytotoxicity and excellent photothermal properties.^35^

In this research, we investigated the controlled release behavior of fluorescent dyes from star polymers crosslinked with thermally-labile azo functionalities. Cleavage of the crosslinks and resultant dye release can be induced by conventional heating and/or NIR irradiation. Biocompatible poly(ethylene glycol)-*block*-poly[(*N*-2-hydroxypropyl) methacrylamide] (PEG-*b*-PHPMA) was synthesized by RAFT polymerization using a PEG-based macro chain transfer agent (macroCTA). Subsequently, a carbodiimide coupling reaction between pendent hydroxyl groups from the PHPMA segment of PEG-*b*-PHPMA and 4,4′-azobis(4-cyanovaleric acid) (**2**) was carried out to form star polymers crosslinked in their cores *via* thermally-labile linkages. This approach of installing thermally-sensitive crosslinks was motivated by our previous work that demonstrated polymers with azo linkages in their backbones are susceptible to heat-induced degradation.^36^ In the current work, after encapsulation of model hydrophobic dyes,^37^ degradation of the star polymers and concomitant release were examined *via* both traditional heating and photothermal heating by NIR irradiation. Further, the effect of ICG incorporation under these conditions was studied. While the goal of this research is not to prepare specific systems for drug delivery, the results suggest NIR-responsive star polymers may hold good potential as smart and effective delivery platforms.

## Results and discussion

### Synthesis of star polymers

**Scheme 1:**
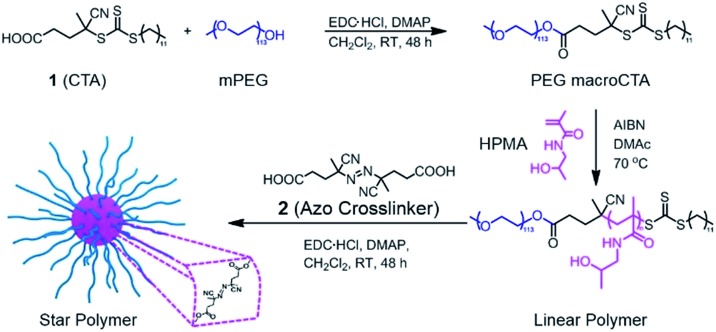
Design and synthesis of thermoresponsive star polymers. Synthesis of PEG-*b*-PHPMA by RAFT polymerization, followed by crosslinking with **2** led to the formation of core-crosslinked star polymers.

Our goal was to synthesize well-defined PHPMA-based star polymers and study their potential for NIR-induced degradation and release. Scheme 1 illustrates the synthetic pathway to the thermally-labile star polymers. RAFT polymerization was utilized to prepare block copolymers with PEG and PHPMA segments. The PEG macroCTA was prepared by a carbodiimide coupling reaction with 4-cyano-4-[(dodecylsulfanylthiocarbonyl)sulfanyl]pentanoic acid (CDTPA, see ESI and Fig. S1†), and subsequently chain extension with HPMA was implemented *via* RAFT polymerization. Various number-average degrees of polymerization (DP) of PHPMA segments were prepared, ranging from 10 to 213 (Table S1†). The compositions of the copolymers were determined by ^1^H NMR spectroscopy, while the absolute number-average molecular weights (*M*_n_) and molar mass dispersities (*Đ*) of the block copolymers were further characterized by gel permeation chromatography (GPC) with multi-angle laser light scattering (MALLS) detection. The molecular weights determined by GPC were in good agreement with those determined by NMR analysis (*M*_n,NMR_) and those predicted based on reaction stoichiometry and monomer conversion (*M*_n,theory_). In all cases, *Đ* values were below 1.2, suggesting efficient chain extension and good polymerization control.

The resulting hydroxyl-containing block copolymers were employed to prepare star polymers by carbodiimide coupling with the difunctional azo compound **2** (Table 1). Somewhat surprisingly, the number of arms (*N*_arm_) per star typically decreased with decreasing arm length, which could be the result of the shorter unimers having fewer hydroxyl groups capable of esterification with the azo-containing diacid compound. On the other hand, addition of **2** to block copolymers with longer PHPMA segments (DP_HPMA_ > 200) resulted in gelation, presumably due to inter-star crosslinking.

**Table 1:** Summary of star polymers synthesized by the carbodiimide coupling of PHPMA block copolymers with compound **2**

Entry	Linear polymer^*a*^	*N*_arm_ ^*b*^	*M*_w,GPC_ ^*c*^ (g mol^–1^)	*Đ* ^*c*^	Size^*d*^ (nm)
1	PEG_113_-*b*-PHPMA_10_	6	4.11 × 10^4^	1.23	27
2	PEG_113_-*b*-PHPMA_24_	11	9.64 × 10^4^	1.45	50
3	PEG_113_-*b*-PHPMA_70_	47	7.88 × 10^5^	1.36	60
4	PEG_113_-*b*-PHPMA_213_	Gelled	n/a	n/a	n/a

^*a*^From Table S1, entries 1–4.

^*b*^Number of arms (*N*_arm_) per star polymer was calculated by the following equation: 
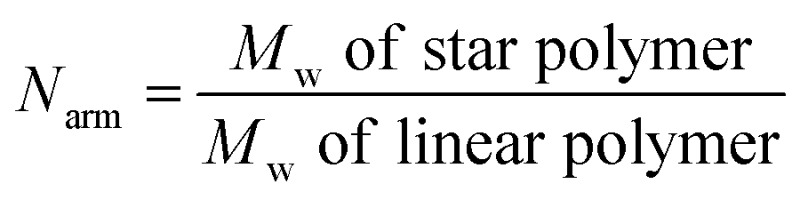
.

^*c*^Determined by GPC-MALLS, assuming d*n*/d*c* values identical to those of the linear polymer precursors (d*n*/d*c* values are listed in Table S1).

^*d*^Determined by DLS in aqueous solution.

**Fig. 1:**
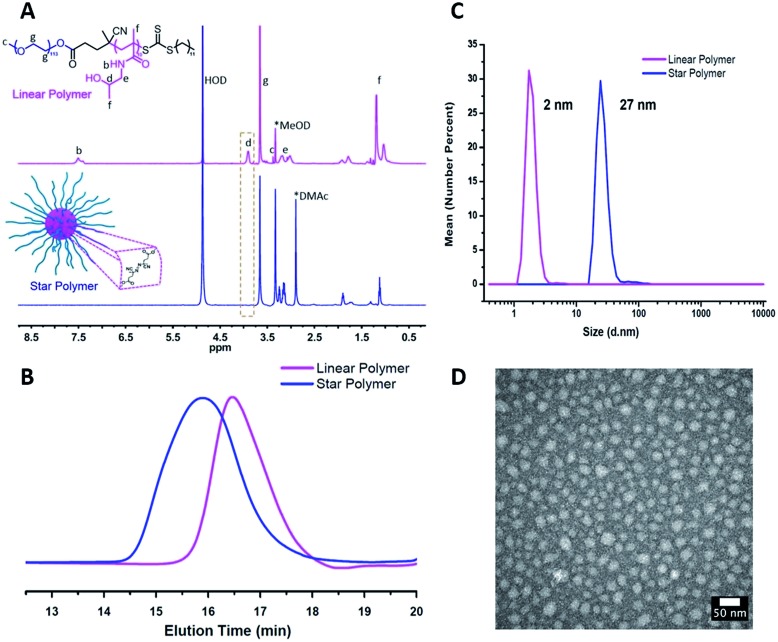
Characterization of star polymers formed from PEG-*b*-PHPMA block copolymers (Table 1, entry 1). (A) ^1^H NMR spectra recorded for linear block copolymer (top) and star polymer (bottom) in MeOD. (B) Gel permeation chromatogram of linear and star polymers by GPC. (C) Number-average hydrodynamic diameter of linear and star polymers measured by DLS. (D) TEM image of the star polymers (stained with uranyl acetate).

^1^H NMR spectroscopy showed the attenuation of the methine resonance of HPMA (CH_3_C ***H*** (OH)CH_2_–, *δ* = 3.8 (a)) after esterification, whereas the resonances from the PEG segments (–OC ***H***_2_C ***H***_2_–, *δ* = 3.6 (g)) were preserved (Fig. 1A). The secondary amide proton signals (–C(=O)N ***H***–CH_2_–, *δ* = 7.5 (b)) and PHPMA backbone signals were also no longer visible, which is consistent with the attenuation of these signals due to the high density and low mobility within the partially desolvated core following esterification.^38,39^ FT-IR spectroscopy further supported successful esterification of the HPMA units *via* a reduction in intensity of the peaks assigned to the hydroxyl groups (*ν* ∼ 3400 cm^–1^) and the appearance of the signal corresponding to the ester carbonyl (*ν* ∼ 1750 cm^–1^, Fig. S2†). As shown in Fig. 1B, a clean peak shift to higher molecular weights was observed for the star polymers by GPC. Dynamic light scattering (DLS) in aqueous media (Fig. 1C, Table 1, entry 1) indicated an increase in hydrodynamic diameter after crosslinking the linear copolymers (∼2 nm) to form the star polymers (∼27 nm). Transmission electron microscopy (TEM) indicated the stars were formed with uniform spherical morphology, and sizes (∼20 nm) were similar to those obtained by DLS analysis (Fig. 1D). Star polymers with larger numbers of arms were characterized in the same manner (Fig. S4 and S5†).

### Dissociation of star polymers by traditional heating

**Fig. 2:**
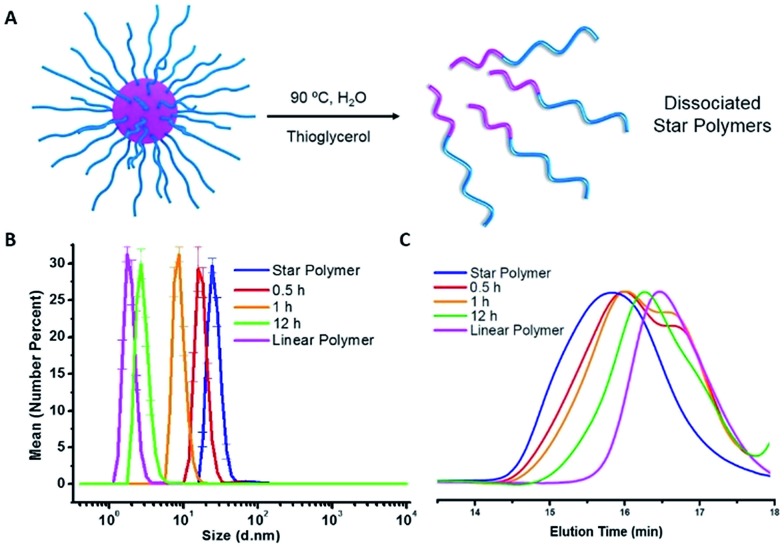
(A) Schematic illustration of heat-triggered star polymer dissociation. (B) DLS size distributions of star polymers during thermal dissociation at 90 °C. (C) GPC eluograms showing star polymer dissociation during heating at 90 °C.

The star polymers were thermally stable and showed no degradation by GPC analysis when stored at room temperature for extended periods (Fig. S3†). However, because the star polymers contained labile azo moieties in their cores (10 h half-life at 69 °C in toluene), we examined the thermally-induced dissociation of the stars at 90 °C in aqueous media (Fig. 2A). Thioglycerol was added to the solution as a radical scavenger to limit undesired intermolecular (re)crosslinking reactions by radical recombination, thereby allowing the dissociation kinetics of the star polymers to be evaluated by DLS and GPC. The thermal degradation behavior of three star polymers (Table 1, entries 1–3) was investigated. Star polymers with larger numbers of arms (Table 1, entries 2 (*N*_arm_ = 11) and 3 (*N*_arm_ = 47)) underwent slow and/or insufficient degradation on heating (Fig. S6 and S7†). Such slow dissociation may be attributed to recombination reactions during degradation, as a result of the high density of azo linkages and insufficient penetration of the radical scavenger within these densely crosslinked star cores. In contrast, smaller star polymers with an average of only 6 arms (Table 1, entry 1) showed considerable degradation under identical conditions. A gradual reduction in size was observed by DLS while heating at 90 °C (Fig. 2B). After 12 h, the average hydrodynamic diameter was reduced to 3 nm, which is slightly larger than that of the linear block copolymers prior to star formation (2 nm). Similarly, the GPC traces indicated gradually decreasing star sizes as a function of heating time (Fig. 2C). The molecular weight of the stars after being treated at 90 °C for 12 h was slightly higher than that of the initial linear polymers prior to crosslinking, presumably due to multiple azo linkages per chain, incomplete scission, and/or a small degree of radical recombination within the star cores. The successful degradation of the star polymers with 6 arms prompted a further investigation of the release behavior of model compounds using this sample.

**Fig. 3:**
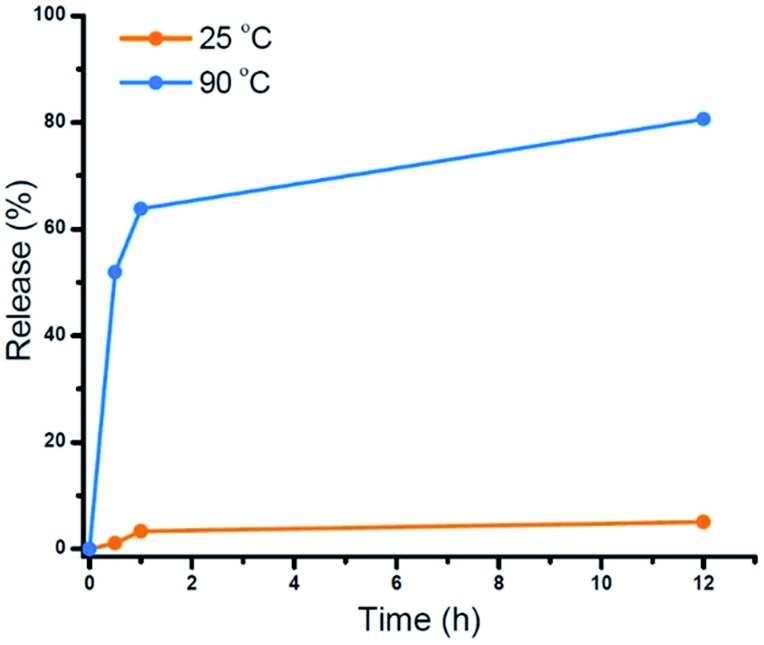
Cumulative NR release profiles from star polymers measured by fluorescence spectroscopy: control (orange, *T* = 25 °C) and heating (blue, *T* = 90 °C).

To demonstrate the potential application of the novel thermally-degradable star polymers as a controlled-release platform, a model compound (Nile red, NR) was encapsulated in the star polymers *via* nanoprecipitation. Briefly, the star polymers and NR were dissolved in tetrahydrofuran (THF), and deionized water was slowly added under vigorous stirring. The mixture was purified by dialysis against deionized water (see ESI†). Fluorescence spectroscopy was employed to determine the content of NR in the stars (Fig. S8†). According to the fluorescence emission spectra, star polymers were successfully loaded with the dye, whereas linear, uncrosslinked block copolymers showed negligible NR stabilization when subjected to the same loading procedure. This result is not surprising, given that only weak interactions are expected between the hydrophobic NR and the hydrophilic PEG-*b*-PHPMA. The release of NR from the loaded star polymers was investigated under similar conditions as the star polymer degradation study. Accumulated dye release caused by dissociation of the star polymer was monitored by the decrease in fluorescence intensity as a function of time.^40^ At 25 °C, negligible dye release (∼5%) from the star polymers was observed. However, more than 60% release was observed after 1 h at 90 °C, and 80% of the NR was released after 12 h at this temperature (Fig. 3). These results indicate the thermally-labile star polymers can be stored at room temperature (Fig. S3†) with triggered degradation attainable by conventional heating. Therefore, we reasoned this nanocarrier could potentially be used for stimulus-induced release.

### Preparation of NIR-sensitive and degradable star polymers

**Fig. 4:**
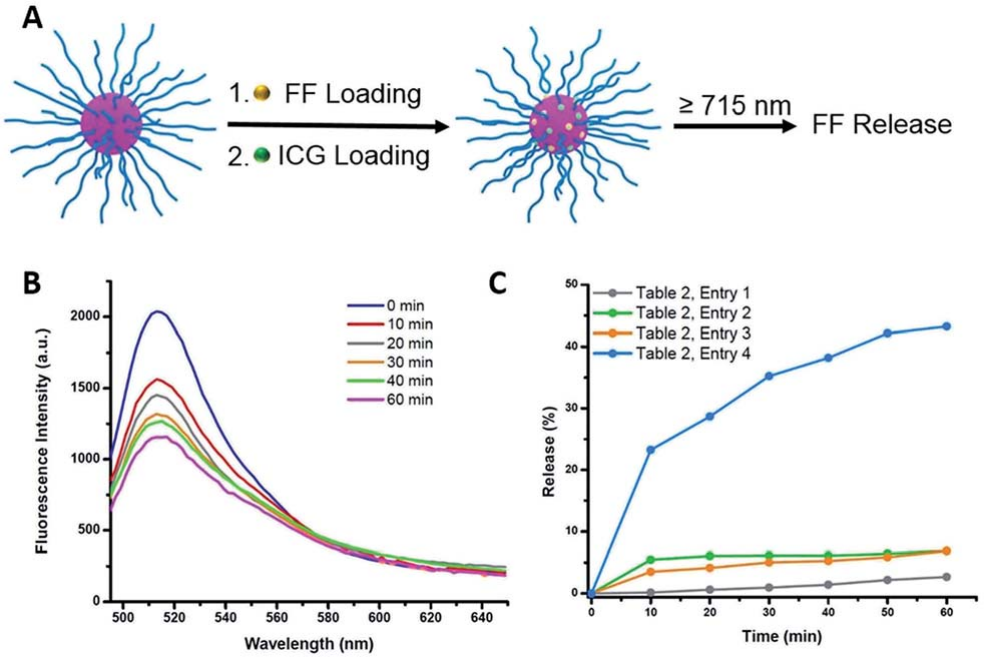
(A) Schematic illustration of FF and ICG encapsulation and NIR triggered FF release *via* photothermal effect. (B) Fluorescence emission spectra (excited at 430 nm) of ICG and FF loaded star polymers under NIR irradiation for varying times. (C) Cumulative release profiles of FF measured by fluorescence intensity changes under different conditions.

Indocyanine green (ICG) is a well-known photosensitizer,^41^ and as such it would be expected to promote decomposition of the azo-groups in the star polymers due to heating by the photothermal effect during NIR irradiation. With this in mind, studies of the photoinduced thermal degradation and triggered release were carried out. When star polymers contained only encapsulated NR, fluorescence intensities remained consistent for 8 h under ambient temperature in the dark (Fig. S10,† blue). However, when both ICG and NR were embedded within the stars, quenching was observed (Fig. S10,† green) which precluded the determination of drug release kinetics *via* fluorescence spectroscopy. Therefore, an additional hydrophobic dye, furan-modified fluorescein (FF), was prepared (Fig. S11†). When FF was encapsulated in the star polymers in conjunction with ICG (Fig. 4A), fluorescence was observed (Fig. S10,† orange), and any remaining non-encapsulated dye could be readily removed by centrifugation and filtration. Visible and NIR absorbance measurements (*λ* = 400–900 nm) of the supernatant were utilized to characterize the ICG loading in the polymer star. As shown in Fig. S9B,† the FF-loaded polymer stars (without ICG) showed no absorption from 550–900 nm, while free ICG exhibited characteristic peaks at *λ* = 700 and 778 nm.^42^ The absorption curve of the ICG-loaded star polymer was slightly red-shifted as compared to that of the free ICG in water, most likely due to the aggregation of ICG in the core.^42^ To further quantify the ICG content of the FF-loaded star polymers, a calibration curve of absorbance *vs.* concentration was obtained using known concentrations of free ICG (*λ*_max_ = 778 nm), as shown in Fig. S9C.† The encapsulation efficiency was determined to be 28% and the loading capacity was 11% for ICG (see ESI, eqn (S1) and (S2)†).

### Photothermally-triggered release

Ground-state ICG can be promoted to the excited state through absorption of a photon of an appropriate energy (*i.e.*, NIR). The molecule will quickly relax to its ground state, releasing this energy *via* either fluorescence or internal conversion (*i.e.* photothermal effect), the latter of which may contribute to localized heating. Therefore, dissociation of the ICG-containing star polymers under NIR irradiation for controlled release of model compounds was examined (Fig. 4A). Briefly, ICG and FF dye were successfully encapsulated by a nanoprecipitation technique (see ESI†). We postulated that the heat generated by NIR irradiation of ICG will lead to the cleavage of the azo bonds within the cores of the star polymers, resulting in release of the FF dye into solution. Therefore, the NIR-triggered release was studied by irradiating solutions of star polymers containing both FF and ICG (*λ* ≥ 715 nm, 1.5 W cm^–2^). The extent of dye release was determined through a decrease in fluorescence intensity at *λ*_max_ = 515 nm (*λ*_exc_ = 430 nm), resulting from precipitation of the hydrophobic dye that was no longer stabilized within the star core (Fig. S12†). In the first 10 min of NIR irradiation, 23% of the dye was released (Fig. 4C, Table 2, entry 4). After further irradiation (*λ* ≥ 715 nm, 1 h), approximately 43% of the original fluorescence intensity was diminished (Fig. 4B). These results indicate that FF release during NIR irradiation is incomplete, presumably due to trapping of FF through recombination of cyanoalkyl radicals or a reaction between the formed radicals and FF in the absence of radical scavengers.

**Table 2:** Summary of photothermally-triggered release conditions

Entry^*a*^	ICG incorporation^*b*^	Dissociation conditions	FF release in 1 h (%)
1	With ICG	25 °C, in the dark	<5
2	With ICG	45 °C, in the dark	<5
3	Without ICG	NIR^*c*^	<5
4	With ICG	NIR	43

^*a*^From Fig. 4C.

^*b*^FF-Loaded star polymer solution with or without ICG.

^*c*^Temperature is between 25 and 45 °C (see Fig. S13).

To unequivocally credit the liberation of FF to the localized heat generated by ICG under NIR irradiation, variables were systematically removed, and the results were examined. First, release from star polymers with encapsulated ICG and FF was studied in the dark (*i.e.*, in absence of NIR trigger). Without NIR irradiation at 25 °C, negligible FF release (<5%, for 1 h) was observed, indicating the star polymers are stable under ambient temperature (Table 2, entry 1). When the temperature was elevated to 45 °C, there was still limited FF release (<8%, Table 2, entry 2), suggesting that the nanocarriers are stable in the absence of NIR irradiation due to a lack of azo bond dissociation. The stability of the star polymers was further investigated under NIR irradiation in the absence of ICG (*i.e.*, no photosensitizer). While the bulk solution temperature rose by a few degrees, insignificant FF release was detected over 1 h (Table 2, entry 3). Irradiated solutions of ICG-encapsulated star polymers showed slightly higher solution temperature increases (*T* = 45 °C) than those of star polymers without ICG (Fig. S13†); however, as mentioned previously, no significant release is observed in this bulk temperature range. The results of these control experiments indicate a locally elevated temperature in the core of the star polymers due to photosensitization of ICG. As a result, effective dye release was found only in ICG-loaded star polymers under NIR irradiation, where temperatures surrounding the azo moieties are sufficiently high to cause appreciable bond dissociation. Although the actual temperature at the star polymer cores upon NIR irradiation could not be verified, the results indicated that the local temperature induced by the photothermal effect of ICG must be much higher than 45 °C in order to break the azo linkages and release the cargo.

## Conclusions

In this report, we developed responsive azo-containing star polymers by combining RAFT polymerization and carbodiimide coupling reactions. This approach resulted in water-soluble particles that were uniform in size and displayed stability in highly dilute solutions. The star polymers were structurally stable up to temperatures of 45 °C in the absence of irradiation, but would rapidly degrade under elevated temperatures (*T* = 90 °C). These results provided evidence those polymers may be used as nanocarriers for temperature-triggered release. This approach was further extended to include heat generated from the interaction of NIR irradiation and a photosensitizer *via* the photothermal effect. ICG was encapsulated as a NIR-responsive photosensitizer in order to generate site-specific heating and induce bond cleavage. Effective release during NIR irradiation was observed only for the star polymers that contained ICG. In contrast, removal of either of the essential components [*i.e.*, the photosensitizer (ICG) or the energy source (NIR)] resulted in minimal disturbance to the polymeric carriers (<8% release), indicating the nanoparticles can be stored under ambient conditions. Combining the results obtained with the major benefits of NIR irradiation (*e.g.*, mild energy source and deep tissue penetration), we anticipate this research can provide an efficient strategy for remote and on-demand photothermal therapy with concomitant release of encapsulated cargo.
