# Supplementary material for: Near-IR-induced dissociation of thermally-sensitive star polymers
Source: Chem Sci. 2016 Dec 13;8(3):1815–21. doi: 10.1039/c6sc04650a (PMC5396554; doi:10.1039/c6sc04650a)
Supplement: Supplementary file 1 [file SC-008-C6SC04650A-s001.pdf]

Electronic Supplementary Material (ESI) for Chemical Science.
This journal is © The Royal Society of Chemistry 2016

Supporting Information for

Near-IR-Induced Dissociation of Thermally-Sensitive Star Polymers

Yuqiong Dai,[a] Hao Sun,[a] Sunirmal Pal,[a] Yunlu Zhang,[b] Sangwoo Park,[a] Christopher P. Kabb,[a] Wei David Wei,[b] Brent S. Sumerlin*,[a]

[a]*George & Josephine Butler Polymer Research Laboratory, Center for Macromolecular Science & Engineering, Department of Chemistry, University of Florida, PO Box 117200, Gainesville, FL 32611-7200, USA. Email: sumerlin@chem.ufl.edu; Fax: +1 352 392 9741*

[b]*Department of Chemistry and Center for Nanostructured Electronic Materials, University of Florida, PO Box 117200, Gainesville, FL 32611-7200, USA.*

Materials. 4-Cyano-4-[(dodecylsulfanylthiocarbonyl)sulfanyl]pentanoic acid (**1**) was prepared as previously reported.[1] Poly(ethylene glycol) monomethyl ether (mPEG 5k, M_n = 5000 g·mol^{-1}), 4,4′-azobis(4-cyanovaleric acid) (**2**, 98%), 4-(dimethylamino)pyridine (DMAP, 99%), Furfurylamine (≥ 99%), Fluorescein, 1,3,5-trioxane, and 2,2′-azobisisobutyronitrile (AIBN) were purchased from Sigma-Aldrich. 1-(3-Dimethylaminopropyl)-3-ethylcarbodiimide hydrochloride (EDC·HCl, 97%) was purchased from Combi-Blocks. Nile red (NR) was purchased from TCI, and indocyanine green (ICG) was purchased from Acros Organics. Deuterated chloroform ($CDCl_3$, 99.8%)

was purchased from Cambridge Isotopes. *N*-(2-Hydroxypropyl)methacrylamide (HPMA) was synthesized as previously reported.[2] Diethyl ether, *N,N*-dimethylacetamide (DMAc), and dichloromethane were purchased from Sigma Aldrich. AIBN was recrystallized from methanol, and all other chemicals were purchased with the highest available purities and used as received.

Instrumental and Characterization. ^{1}H NMR spectra were recorded on a Varian Inova2 500 MHz NMR spectrometer, using the residual solvent signal as reference. Fluorescence and UV-Vis spectra were obtained on a Molecular Devices SpectraMax M2 multimode microplate reader at 25 °C. Measurements were conducted on clear (absorbance) or black (fluorescence) 96-well polypropylene microplates (Greiner Bio-One). Molecular weights and molecular weight distributions were determined *via* size exclusion chromatography with multi-angle laser light scattering (SEC-MALLS) in DMAc with 50 mM LiCl at 50 °C and a flow rate of 1.0 mL/min (Agilent isocratic pump, degasser, and autosampler; ViscoGel I-series 10 μm guard column and two ViscoGel I-series G3078 mixed bed columns, with molecular weight ranges 0−20 × 10^3 and 0−10 × 10^6 g/mol, respectively). Detection consisted of a Wyatt Optilab TrEX refractive index detector operating at 658 nm and a Wyatt miniDAWN Treos light scattering detector operating at 659 nm. Absolute molecular weights and polydispersities were calculated using Wyatt ASTRA software. Dynamic light scattering (DLS) measurements were recorded on a Zetasizer Nano ZS, (Malvern Instrument Ltd., U.K.) equipped with a He−Ne laser beam operating at 633 nm at 25 °C. Samples were prepared in pure water and filtered through a 0.45 μm nylon

syringe filter prior to analysis. Each measurement was repeated six times to obtain an average value. Transmission electron microscopy (TEM) was conducted on a Hitachi H7000 microscope operating at 100 kV. A Formvar coated 200-mesh Cu grid that was freshly glow discharged (Pelco easiGlow™, Ted Pella, Inc.) was placed onto a drop of sample solution for 30 sec and wicked off with filter paper. Uranyl acetate (0.5% aqueous solution) was used as a negative stain. Infrared spectra were collected on a Thermo Nicolet 5700 FT-IR spectrometer equipped with a single bounce, diamond-stage attenuated total reflectance (ATR) accessory. In all the photothermal experiments, a 300W Xenon lamp (Newport) was used with a longpass filter ($\lambda \geq 715$ nm, 1.5W/cm^2). Substrates were placed in a glass vial and the solution temperature was measured every 10min during irradiation.

Synthesis of PEG-Based Macro Chain Transfer Agent (macroCTA). As modified from a previously reported procedure,[3] a solution of mPEG 5k (5.0 g, 1.0 mmol) and **1** (5.3×10^2 mg, 1.3 mmol) in dichloromethane (15 mL) was cooled in an ice bath. A solution of EDC·HCl (2.6×10^2 mg, 1.3 mmol) and DMAP (14 mg, 1.2×10^{-1} mmol) was added dropwise. The reaction was stirred overnight at room temperature. The macroCTA was precipitated into cold diethyl ether (2 × 100 mL) and collected. The product was then dissolved in dichloromethane (100 mL) and washed with distilled water (3 × 70 mL). The organic layer was collected, and the residual water was removed by treating it with anhydrous Na_2SO_4. The mixture was then filtered and concentrated under reduced pressure. Finally, the solution was precipitated in cold diethyl ether. The obtained product

was dried in a vacuum oven at room temperature for 48 h. 3.2 g (yield = 60%) of product was obtained.

Synthesis of Block Copolymers. An example RAFT polymerization of HPMA is as follows: HPMA (7.0×10^{2} mg, 5.0 mmol), PEG macroCTA (1.3 g, 2.5×10^{-1} mmol), AIBN (16 mg, 1.0×10^{-1} mmol), 1,3,5-trioxane (1.5×10^{2} mg, 1.7 mmol), and DMAc (6.0 mL) were placed in a 25 mL Schlenk flask ([HPMA]/[MacroCTA]/[AIBN] = 20/1/0.2). Oxygen was removed over the couse of three freeze-pump-thaw cycles. At the end of the final cycle, the Schlenk flask was filled with N_2 and placed in a preheated oil bath at 70 °C. Samples were taken periodically by syringe to determine molecular weight and dispersity (*Đ*) by GPC. Monomer conversion was confirmed by ^{1}H NMR spectroscopy. The final polymer was isolated by precipitating in diethyl ether. The resulting polymers were dried in a vacuum oven overnight. All polymerizations were quenched at 50 % conversion of HPMA. Detailed information is listed in Table S1.

Star Formation. A solution of the block copolymer and **2** ([-COOH]/[-OH] = 2/5) in dichloromethane (15 mL) was cooled in an ice bath. A solution of EDC·HCl (3 equiv. relative to [**2**]) and DMAP (0.1 equiv. relative to [EDC·HCl]) was added dropwise. The reaction was stirred overnight at room temperature. The solution was then precipitated into cold diethyl ether (4 × 100 mL), and the resulting product was dried in a vacuum oven for 48 h. Tangential flow filtration (TFF) with a 70 kg/mol molecular weight cut off (MWCO) membrane was used to further purify the star polymers.

Degradation of Star Polymers by Traditional Heating Method. An aqueous solution

of star polymers (2.5 mg/mL) and the radical scavenger (thioglycerol, 10 equiv. relative to azo moieties) was heated to 90 ºC in an oil bath. Samples were taken periodically for GPC and DLS characterization.

Dye Encapsulation. Nile red (NR) was encapsulated into the stars by a nanoprecipitation method. Briefly, NR (1.0 mg) and PEG_{113}-*b*-$PHPMA_{10}$ block polymer (5.0 mg) were dissolved in 1 mL THF, and then deionized H_2O (10 mL) was quickly added under vigorous stirring. The solution was then dialyzed against water (MWCO = 3,500 g/mol) at room temperature for 6 h. The dye-encapsulated stars were further purified to remove non-encapsulated dye by filtration using a 0.45 μm nylon syringe filter. The furan-fluorescein dye was encapsulated under the same procedure.

Synthesis of Furan-Modified Fluorescein (FF). Fluorescein (1.0 g, 3.0 mmol) was dissolved in DCM (9 mL) and DMF (6 mL). Then, EDC·HCl (1.3 g, 6.6 mmol) and DMAP (0.9×10^{-2} g, 0.8 mmol) were added to the solution. The mixture was stirred for 3 h, followed by the dropwise addition of furfuryl amine (0.7 mL, 7.5 mmol). Thereafter, the reaction was allowed to stir for 24 h. After the reaction, the solvent was removed by rotary evaporation, and the residue was dissolved in ethyl acetate (50 mL). The organic solution was then washed first with HCl solution (1.0 M) and then brine. The organic layer was collected and dried over Na_2SO_4. After removing the solvent under vacuum, the product was collected as a yellow solid (0.7 g, 56 %).

ICG Encapsulation. ICG (1 mg) was dissolved in the dye-loaded star polymer solution in the dark, and the resulting solution was stirred for 2 h. To remove the excess ICG, the

aqueous mixture was spin-filtered three times with 1.2 mL of deionized water using Amicon Ultra 1.5 mL centrifugal filter units (10 kDa MWCO) at 12,000 rpm for 20 min. The ICG calibration curve was obtained using ICG aqueous solutions of known concentration. The ICG weight fraction and encapsulation efficiency were calculated by comparing the absorbance intensity of ICG in the supernatant (10 times diluted). The absorption spectra in the region from 400 nm to 900 nm were also used to characterize the loading property of ICG for the star polymers.

NIR-Triggered FF Release *via* ICG Photothermal Effect. Samples of dye-loaded star polymer aqueous solutions (3 mL) with different ICG concentrations were placed in glass vials and irradiated with NIR light (λ = 715 nm) for 1 h. During the irradiation period, samples were removed periodically and the solution temperature was measured at the same time. The temperature profiles are shown in Fig. S8.

Supplemental Figures

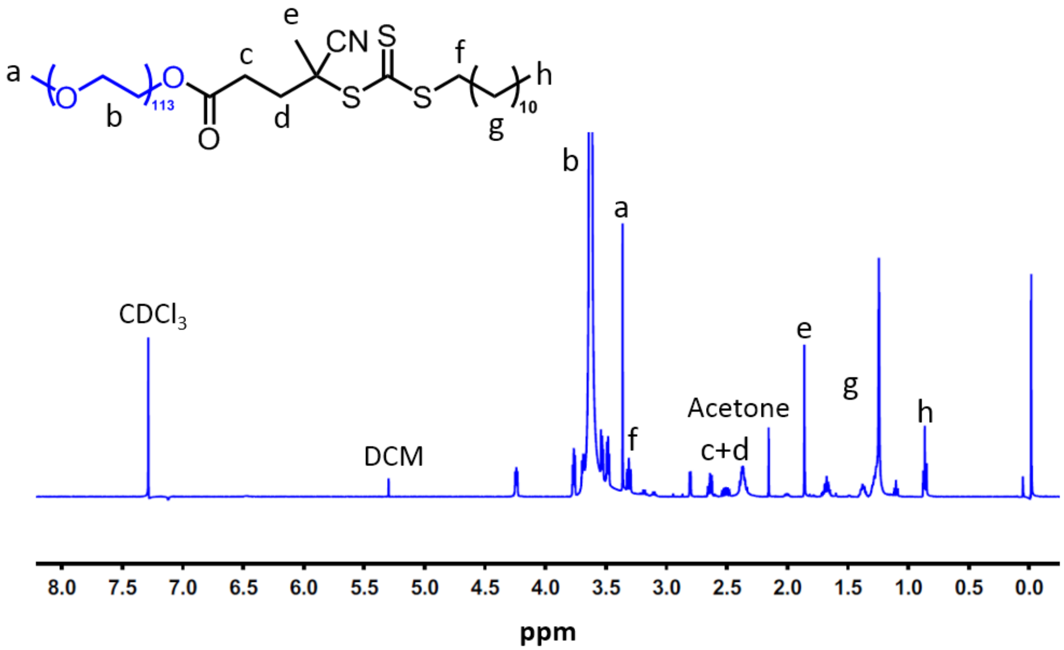

Fig. S1. ^{1}H NMR spectrum of PEG macroCTA in $CDCl_3$ at 25 ºC.

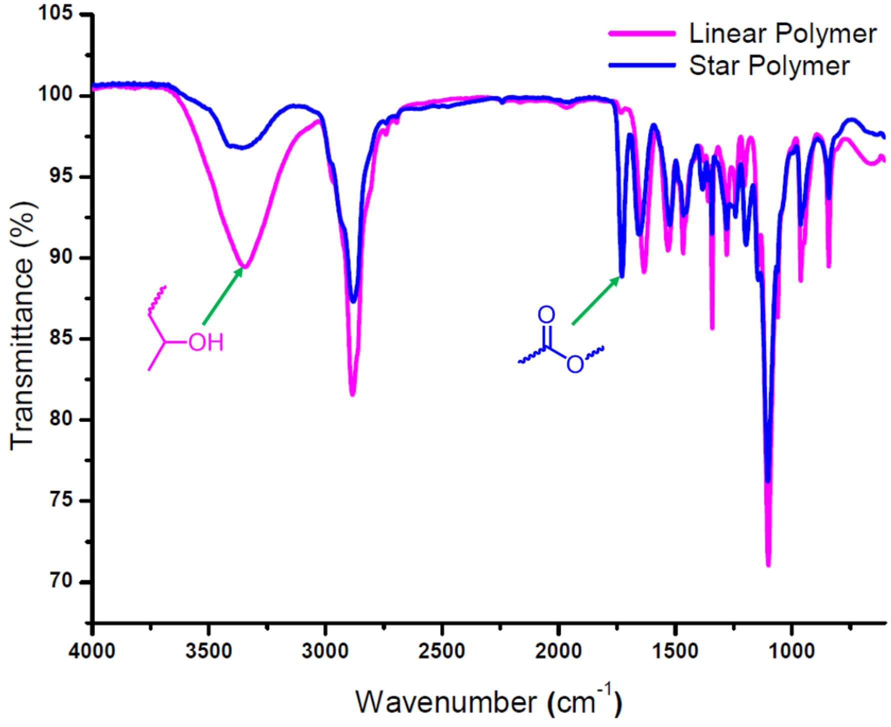

Fig. S2. FT-IR spectra of linear polymer (magenta) and star polymer (blue). Arrows indicate hydroxyl and ester functional groups.

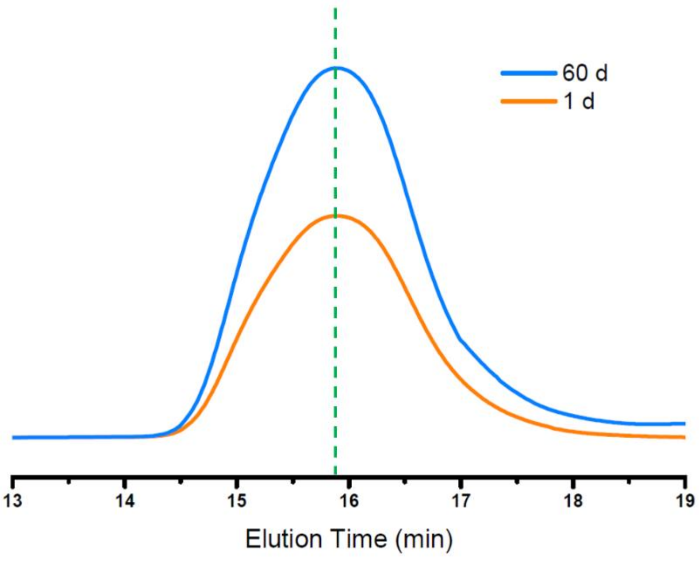

Fig. S3. GPC eluograms of star polymers (Table S1, entry 1) after 1 day (orange) and 60 days later (blue). No significant changes were observed, indicating the high stability of the star polymers at room temperature.

Decomposition rate constant (k_d) of an azo initiator and half-life time ($t_{1/2}$) are defined as:[4]

$$k_d = A\exp(-\frac{E_d}{RT}) \quad \text{(S-1)}$$

$$t_{1/2} = (\ln 2)/k_d \quad \text{(S-2)}$$

For compound **2**, Where, A is the pre-exponential factor, E_d is the activation energy (for dissociation, kJ/mol), R is the gas constant (8.314 J/K·mol). For compound **2**, E_d = 132 kJ/mol, ln (A) = 36/s. At 25 ºC (T = 298.15 K), $t_{1/2}$≈ 249 d in *N,N*-dimethylformamide. This estimation indicates that the dissociation of the star polymers would be negligible at room temperature over extended time periods.

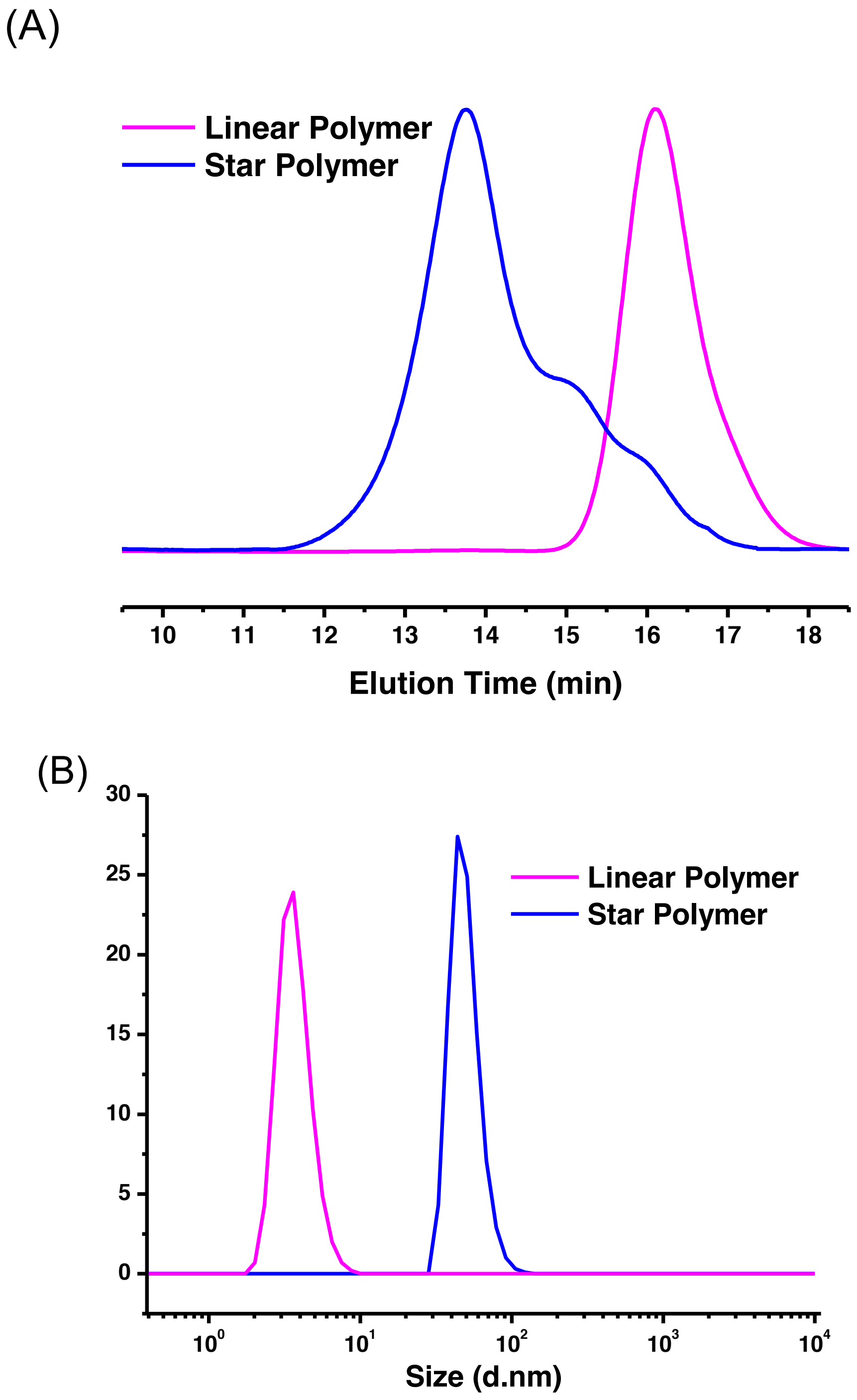

Fig. S4. Characterization of star polymers with DP_{HPMA} = 24 (Table S1, entry 2). (A) GPC eluogram and (B) particle size analysis by DLS.

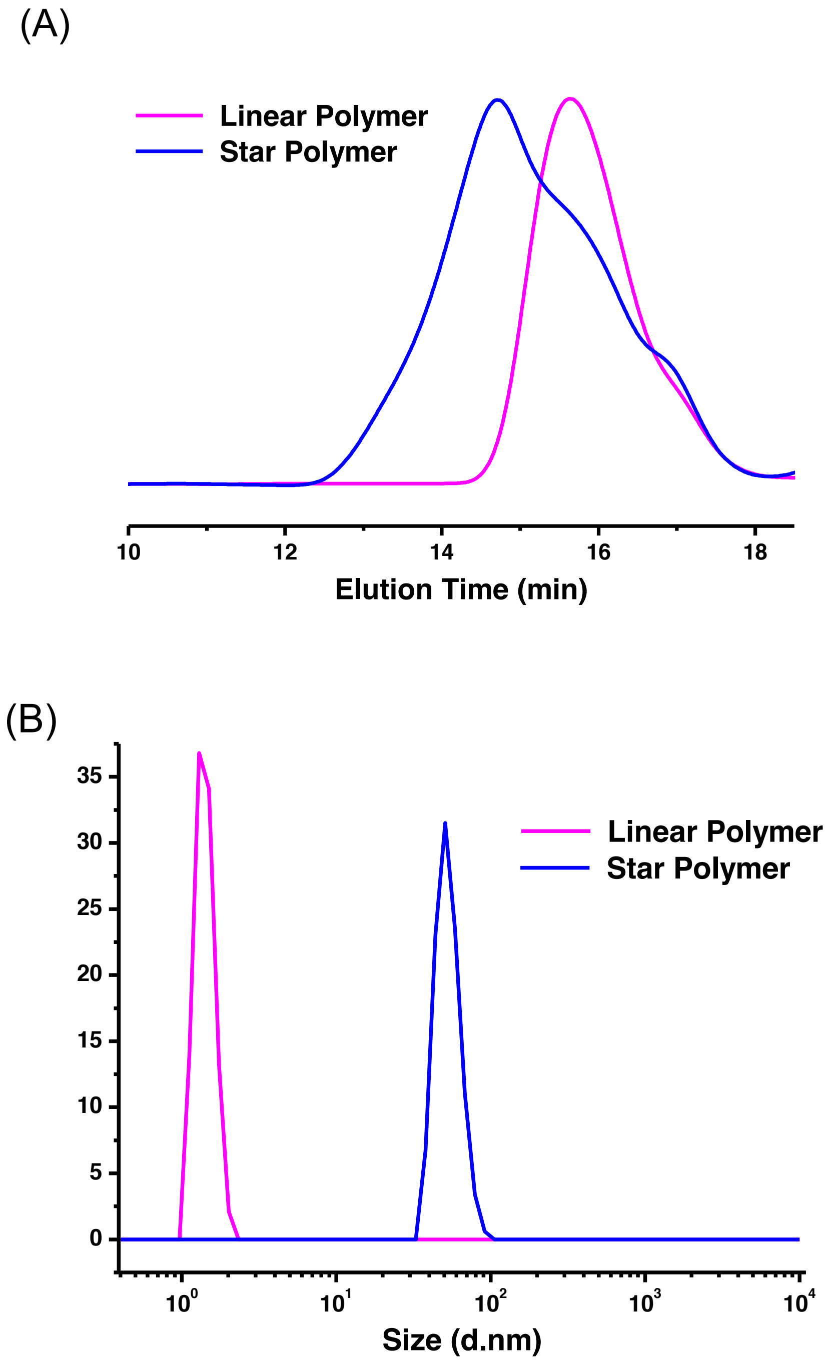

Fig. S5. Characterization of star polymers DP_{HPMA} = 70 (Table S1, entry 3). (A) GPC eluogram and (B) particle size analysis by DLS.

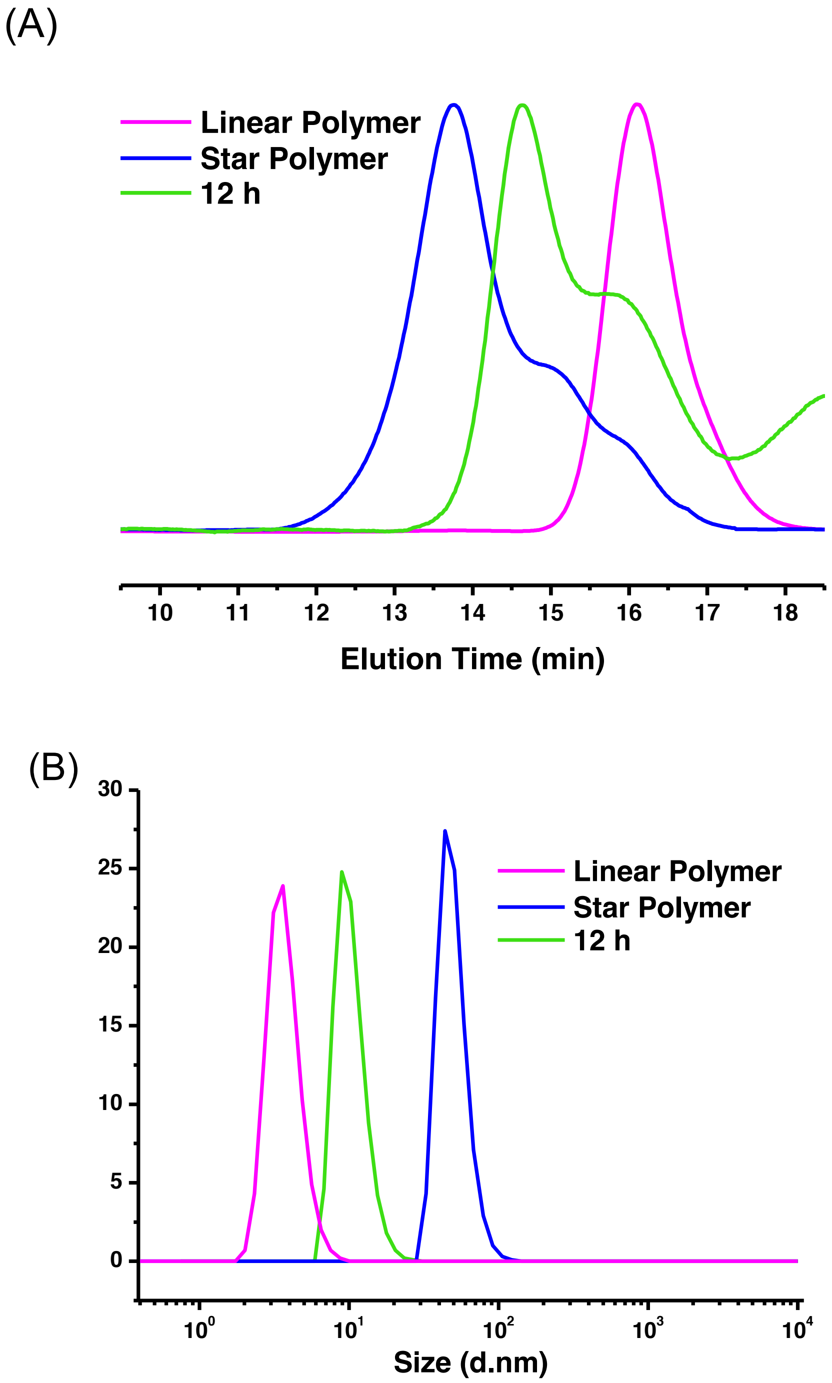

Fig. S6. Thermal degradation of star polymers DP_{HPMA} = 24 (Table S1, entry 2) in presence of radical scavenger at 90 ºC. (A) GPC eluogram and (B) particle size analysis by DLS.

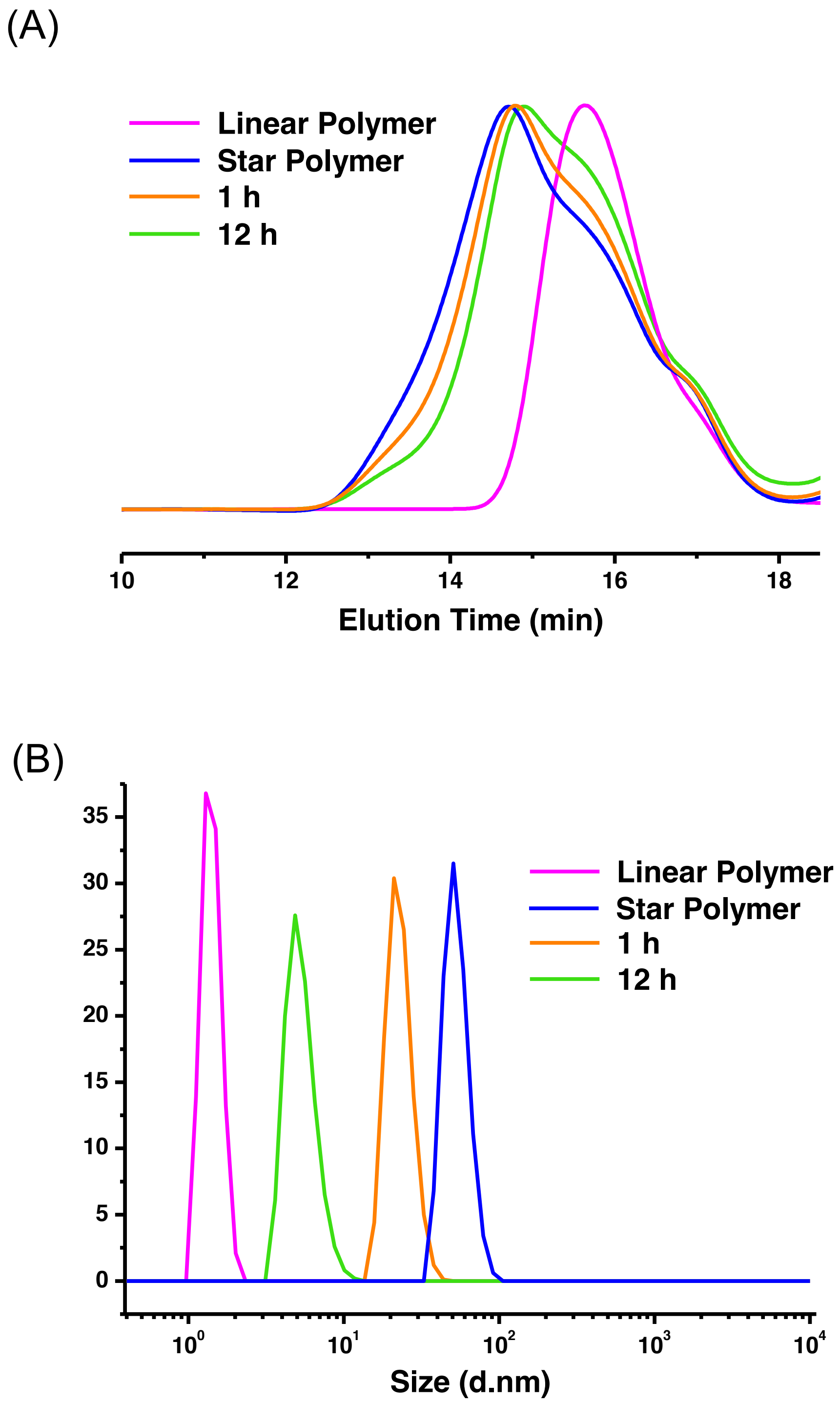

Fig. S7. Thermal degradation of star polymers DP_{HPMA} = 70(Table S1, entry 3) in the presence of radical scavenger at 90 ºC. (A) GPC eluogram and (B) particle size analysis by DLS.

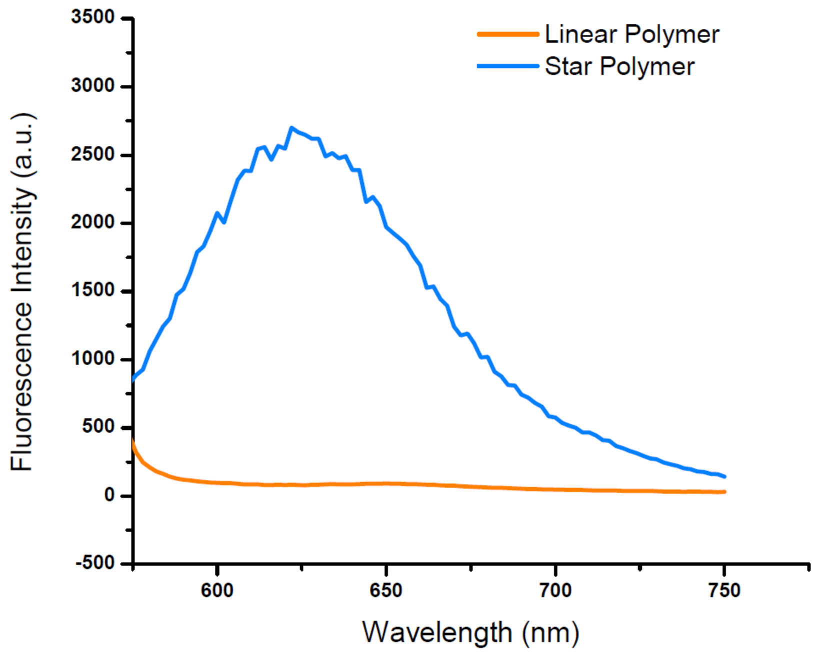

Fig. S8. Fluorescence emission spectra (λ_{exc} = 530 nm) of NR loaded star polymers (blue) and linear polymer (orange).

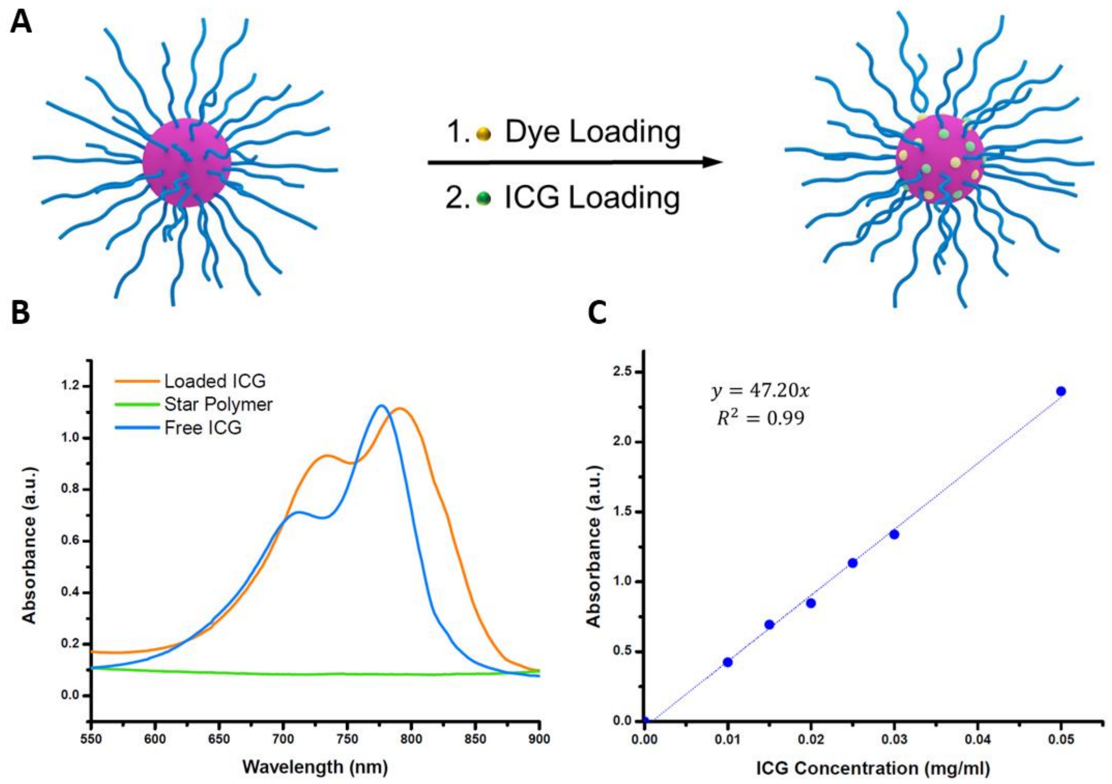

Fig. S9 (A) Schematic illustration of FF and ICG encapsulation. (B) UV-Vis absorption spectra of FF- and ICG-loaded star polymers (orange), FF-loaded star polymers (green) and free ICG in H_2O (blue). (C) Calibration curve using ICG aqueous solutions with known concentrations at 778 nm.

$$\text{Encapsulation efficiency (\%)} = \frac{\text{Mass of total ICG - Mass of ICG in supernatant}}{\text{Mass of total ICG}} \times 100 \qquad \text{(S-3)}$$

$$\text{ICG weight fraction (\%)} = \frac{\text{Mass of ICG in star polymers}}{\text{Mass of star polymers}} \times 100 \qquad \text{(S-4)}$$

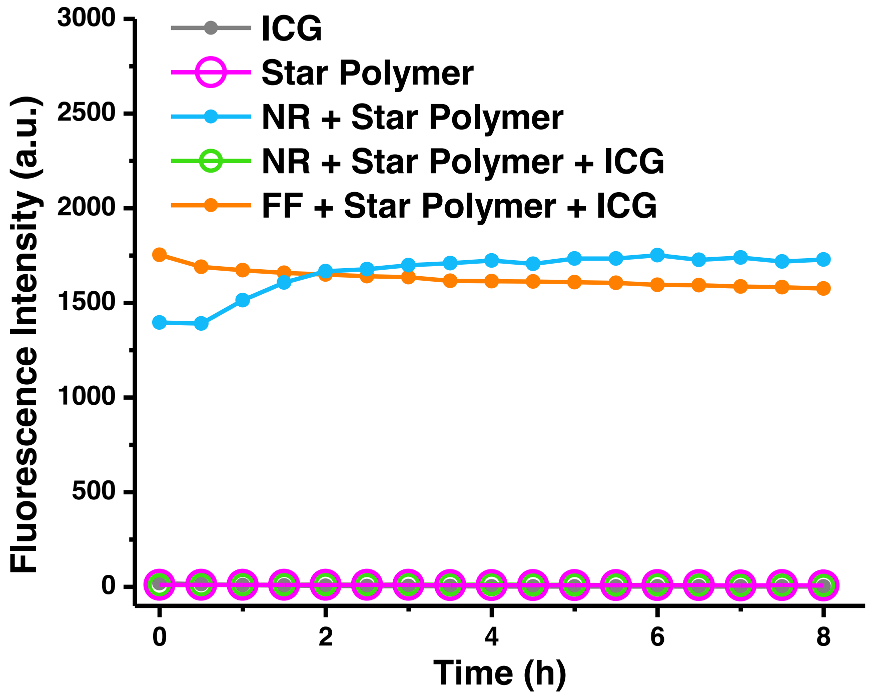

Fig. S10. Fluorescence intensity kinetics of NR and FF dyes in the presence or absence of ICG in the dark.

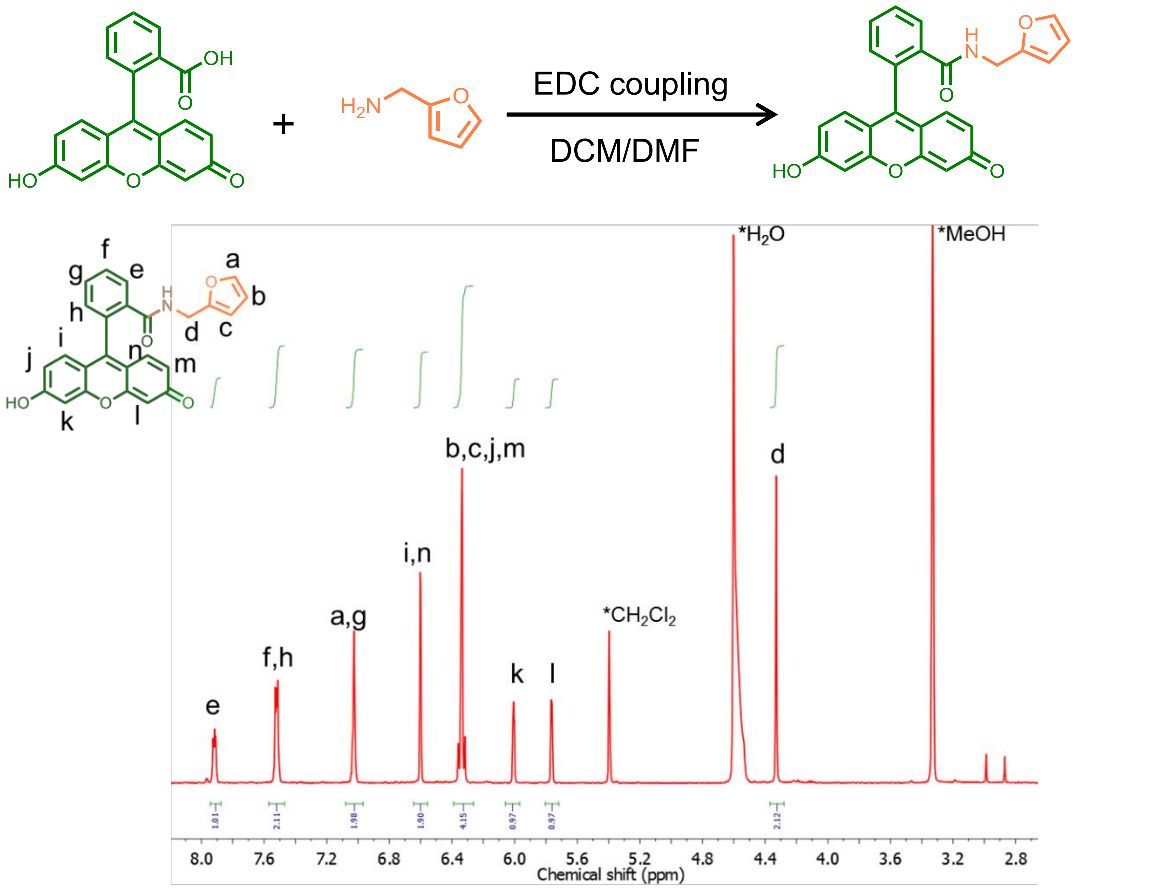

Fig. S11. ^{1}H NMR spectrum of furan-modified fluorescein (FF) in CD_2Cl_2:MeOD (V/V = 1:1) at 25 ºC.

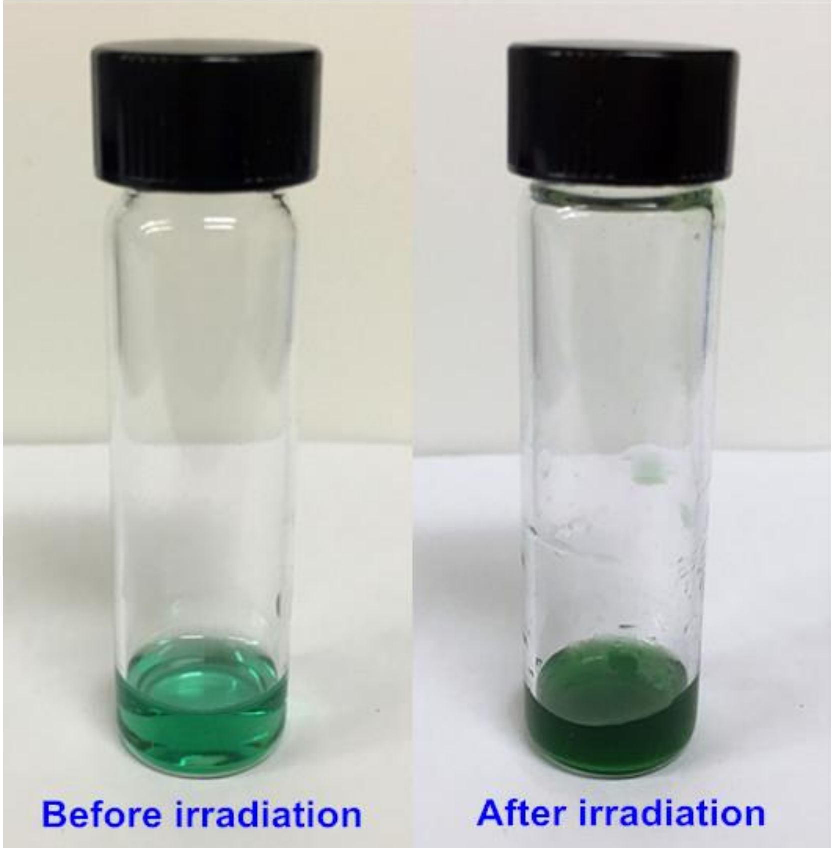

Fig. S12. Digital photos of the FF and ICG loaded star polymer solution before and after NIR irradiation.

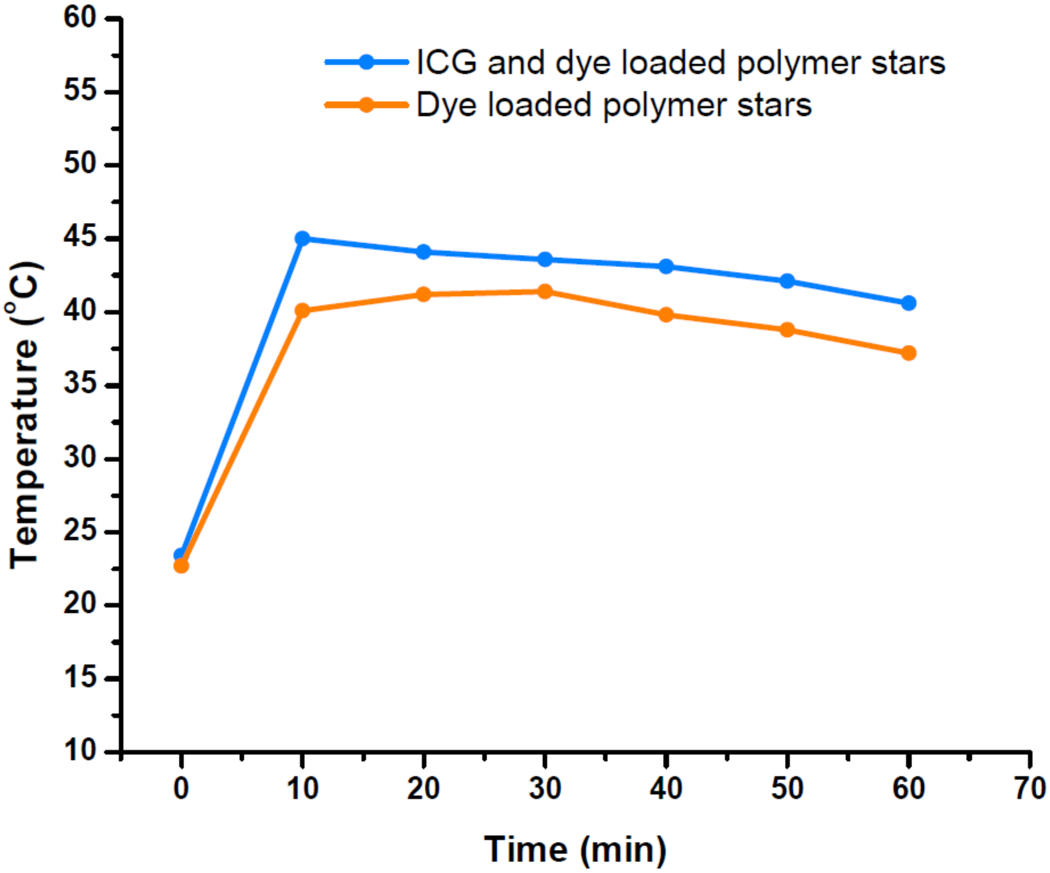

Fig. S13. Temperature profiles of the FF-loaded star polymer solution in the presence or absence of ICG under NIR irradiation.

Supplemental Tables

Table S1. Summary of PEG-*b*-PHPMA block copolymers.

Entry[a]	DP_{HPMA}[a]	$M_{n,NMR}$ ($g{\cdot}mol^{-1}$)[b]	$M_{n,theory}$ ($g{\cdot}mol^{-1}$)[c]	$M_{n,GPC}$ ($g{\cdot}mol^{-1}$)[d]	d*n*/d*c*[d]	$Đ$[d]
1	10	6.82×10^3	6.82×10^3	6.12×10^3	0.0534	1.15
2	24	8.82×10^3	8.97×10^3	8.05×10^3	0.0727	1.12
3	70	1.54×10^4	1.61×10^4	1.38×10^4	0.0802	1.21
4	213	3.64×10^4	4.12×10^4	4.83×10^4	0.0893	1.27

Reaction conditions: [HPMA]/[macroCTA]/[AIBN] = x/1/0.2 (x = 20, 50, 150, and 500 for entries 1-4, respectively); T = 70 °C; [macroCTA] = 0.04 M; all the reactions were stopped at 50% monomer conversion. [a] DP of PEG was 113; [b] Calculated by comparing peak intensities between PEG backbone ($-OC\boldsymbol{H}_2C\boldsymbol{H}_2-$) and HPMA side chains ($CH_3C\boldsymbol{H}(OH)CH_2-$); [c] $M_{n,theory}$ = $[HPMA]_0/[macroCTA]_0$ × conversion × MW_{HPMA} + $MW_{macroCTA}$; [d] Determined by SEC-MALLS in DMAc by 100% mass recovery technique (ASTRA software, Wyatt Technology).

References

1. G. Moad, Y. Chong, A. Postma, E. Rizzardo and S. H. Thang, *Polymer*, 2005, **46**, 8458-8468.
2. B. S. Tucker, S. G. Getchell, M. R. Hill and B. S. Sumerlin, *Polym. Chem.*, 2015, **6**, 4258-4263.
3. S. Maji, G. Vancoillie, L. Voorhaar, Q. Zhang and R. Hoogenboom, *Macromol. Rapid Commun.*, 2014, **35**, 214-220.
4. T. Cheikhalard, L. Tighzert and J. P. Pascault, *Macromol. Mater. Eng.*, 1998, **256**, 49-59.
